# Determination of breast cancer prognosis after neoadjuvant chemotherapy: comparison of Residual Cancer Burden (RCB) and Neo-Bioscore

**DOI:** 10.1038/s41416-020-01251-3

**Published:** 2021-02-09

**Authors:** Enora Laas, Julie Labrosse, Anne-Sophie Hamy, Gabriel Benchimol, Diane de Croze, Jean-Guillaume Feron, Florence Coussy, Thomas Balezeau, Julien Guerin, Marick Lae, Jean-Yves Pierga, Fabien Reyal

**Affiliations:** 1 Department of Surgery, Institut Curie, Paris, France; 2 Residual Tumor & Response to Treatment Laboratory, RT2Lab, Translational Research Department, PSL Research University, INSERM, U932 Immunity and Cancer, Institut Curie, Paris, France; 3 Department of Medical Oncology, Institut Curie, Paris, France; 4 Data Office, Institut Curie, 26 rue d’Ulm, 75005 Paris, France; 5 Department of Pathology, Institut Curie, Paris, France; 6 University of Paris Descartes (Paris V), Paris, France

**Keywords:** Breast cancer, Breast cancer, Chemotherapy

## Abstract

**Background:**

To compare RCB (Residual Cancer Burden) and Neo-Bioscore in terms of prognostic performance and see if adding pathological variables improve these scores.

**Methods:**

We analysed 750 female patients with invasive breast cancer (BC) treated with neoadjuvant chemotherapy (NAC) at Institut Curie between 2002 and 2012. Scores were compared in global population and by BC subtype using Akaike information criterion (AIC), C-Index (concordance index), calibration curves and after adding lymphovascular invasion (LVI) and pre-/post-NAC TILs levels.

**Results:**

RCB and Neo-Bioscore were significantly associated to disease-free and overall survival in global population and for triple-negative BC. RCB had the lowest AICs in every BC subtype, corresponding to a better prognostic performance. In global population, C-Index values were poor for RCB (0.66; CI [0.61–0.71]) and fair for Neo-Bioscore (0.70; CI [0.65–0.75]). Scores were well calibrated in global population, but RCB yielded better prognostic performances in each BC subtype. Concordance between the two scores was poor. Adding LVI and TILs improved the performance of both scores.

**Conclusions:**

Although RCB and Neo-Bioscore had similar prognostic performances, RCB showed better performance in BC subtypes, especially in luminal and TNBC. By generating fewer prognostic categories, RCB enables an easier use in everyday clinical practice.

## Background

Neoadjuvant chemotherapy (NAC) is currently administered to patients with locally advanced breast cancers (BC), to BC of poor prognosis (triple-negative and *HER2*-positive tumours, or BC with nodal involvement and/or high proliferation rates), or to early stage BC having an indication of systemic therapy.^1,2^ Beyond increasing breast-conserving surgery rates,^3–5^ NAC enables the evaluation of systemic treatments in vivo, thus making it theoretically possible to discontinue ineffective treatments.^6,7^ Response to NAC also carries important prognostic information. Indeed, patients with pathological complete response (pCR) after NAC were reported to have more favourable long-term outcomes,^8,9^ especially for *HER2*-positive and triple-negative BC (TNBC).^10^ However, a minority of tumours reach pCR after NAC. Depending on definitions of residual disease (ypT0 ypN0 or ypT0/is, respectively), pCR rates vary from 13% (IC95%^12–14^) to 22% (IC95%^21,22^).^10^

Hence, prognostic scores such as RCB (Residual Cancer Burden index),^8^ CPS (Clinical-Pathologic Scoring),^11^ CPS + EG (oestrogen-receptor (E) status and nuclear grade (G))^11^ and Neo-Bioscore^12^ were developed to classify BC patients into different prognostic risk categories after NAC. Thereby, patients considered as having a high risk of relapse can be candidates to further second-line treatments (TDM-1, Capecitabine) or clinical trials. Pathological variables such as lymphovascular invasion (LVI) and tumour-infiltrating lymphocytes**’** (TILs) may also have a prognostic value after NAC.^13,14^ LVI, defined as the presence of tumour cells in lymphatic or blood vessels, was identified as a risk factor of axillary and distant metastasis,^15,16^ associated to higher risks of node involvement, distant metastasis and death.^13,17–19^ High TILs levels on BC biopsy have been associated to high pCR rates in the neoadjuvant setting and to better outcomes in the adjuvant setting.^20–23^

Altogether, although RCB has been suggested as the preferred score,^24^ its prognostic performance has not yet been compared to more recent models such as Neo-Bioscore. Furthermore, despite growing evidence of their impact, whether LVI and/or TILs improve the prognostic performance of scores after NAC remains unknown.

The objective of the present study was to compare the main models that exist to refine prognosis after NAC by evaluating their prognostic performance in a large real-life cohort of BC patients, and to determine whether adding pathological variables improved these scores.

## Methods

### Patients and tumours

We analysed a cohort of 750 female patients with T1–3NxM0 invasive BC (NEOREP Cohort, CNIL declaration number 1547270) treated with NAC at Institut Curie between 2002 and 2012. The cohort included unifocal, unilateral, nonrecurrent, nonmetastatic tumours, excluding T4 tumours (inflammatory, chest wall or skin invasion). All patients included in the cohort received NAC. Patients were treated according to national guidelines. Approved by the Breast Cancer Study Group of Institut Curie, the study was conducted according to institutional and ethical rules concerning research on tissue specimens and patients. Informed consent from patients was not required.

Information on clinical and tumour characteristics were retrieved from medical health records.

ER, PR and *HER2* positivity determination and treatment protocol are detailed in the supplemental material. Based on immunohistochemistry surrogates, pathological subtypes were defined as follows: tumours positive for either ER or PR and negative for *HER2* were classified as luminal; tumours positive for *HER2* were classified as *HER2*-positive BC; tumours negative for ER, PR and *HER2* were classified as triple-negative BC (TNBC).

RCB and Neo-Bioscore were retrospectively reviewed by a single pathologist.

### Prognostic models

pCR was defined as the absence of residual invasive cancer cells in the breast and axillary lymph nodes (ypT0/is +ypN0).^10^

Residual cancer burden (RCB) index was calculated using the web-based calculator (available on internet),^25^ by considering dimensions of the primary tumour, tumour bed cellularity and axillary nodal burden.^8^ RCB is composed of four categories: RCB‐0 (complete pathologic response = pCR), RCB‐I (minimal residual disease), RCB‐II (moderate residual disease) and RCB‐III (extensive residual disease).

Neo-Bioscore^12^ derives from the CPS and CPS + EG scores. According to the American Joint Committee on Cancer (AJCC) guidelines, Neo-Bioscore is calculated by considering for each patient the pretreatment clinical stage and the post-treatment pathologic stage.^26^ Additional points are added in case of ER-negative disease, nuclear grade 3 tumours, and *HER2*-negative tumours. Neo-Bioscore is composed of eight categories (Neo-Bioscore 0–7), of increasingly poor prognosis.

LVI was defined as the presence of carcinoma cells within a finite endothelial-lined space (a lymphatic or blood vessel). Presence or absence of LVI was determined by unstained standard formalin-fixed paraffin-embedded examination. Immunostaining with vascular markers was occasionally performed to rule out invasive carcinoma with shrinkage artefact. Data concerning LVI were extracted from pathologic records by two independent researchers.

TILs levels were evaluated on pretreatment core needle biopsies and post-NAC surgical specimens for the presence of mononuclear cells infiltrate (including lymphocytes and plasma cells, excluding polymorphonuclear leukocytes), following the international TILs Working Group recommendations.^27^ They were evaluated in stroma, within tumour scar border, after excluding areas around ductal carcinoma in situ, tumour zones with necrosis and artefacts, and were scored continuously as the average percentage of stromal area occupied by mononuclear cells. Pre-NAC TILs were described in categories (low: <10%; intermediate: 11–59%; high ≥60%).^14^ Post-NAC TILs were analysed as a continuous variable, as no threshold has yet been determined.

Since RCB and Neo-Bioscore are composed of a different number of risk categories (four risk categories for RCB vs. eight risk categories for Neo-Bioscore, respectively), establishing common risk categories was necessary to compare the two scores. Hence, based on the predicted risk of events, we established three risk categories: low risk (predicted 5-year DFS > 90%, corresponding to RCB-I/pCR and Neo-Bioscore 0–3); intermediate risk (predicted 5-year DFS comprised between 70 and 90%, corresponding to RCB-II and Neo-Bioscore 4–5); and high risk (predicted 5-year DFS < 70%, corresponding to RCB-III and Neo-Bioscore 6–7).

### Study endpoints

Disease-free survival (DFS) was defined as the time from surgery to death, loco-regional recurrence or distant recurrence, whichever occurred first. Overall survival (OS) was defined as the time from surgery to death. Patients for whom none of these events were recorded were censored at the date of their last known contact.

### Statistical analysis

The Akaike Information Criterion (AIC)^28^ was used to compare prognostic performances. AIC determines the best prognostic model from a set of models by selecting the one with the highest likelihood under the constraint of the smallest number of predictors. The lowest AIC value corresponds to the best model.

Discrimination (i.e. whether the relative ranking of individual predictions is in the correct order) was evaluated using the concordance index (C-Index).^29^ C-Index is the probability that given two randomly selected patients, the patient with the most pejorative outcome will in fact have the most pejorative predicted outcome. A C-index value of 0.9–1.0 corresponds to an excellent discriminative power, whereas a C-Index value of 0.5 corresponds to a worthless test. Its discriminative power is considered poor from 0.6 to 0.7, fair from 0.7 to 0.8 and good from 0.8 to 0.9. C-Index can be used for censored data. A bootstrap method with 500 resample was used to determine confidence intervals.

Calibration (i.e. the relationship between outcomes observed and predicted probabilities) was evaluated with graphical representations (calibrations curves). By definition, a well-calibrated risk score or prediction rule attributes the correct probability of event to all predicted risk levels. A poorly calibrated rule under- or over-predicts the probability of events. Calibration applied on survival analysis are particular since observations are events. Well-calibrated models have an intercept α of zero and a slope β of 1. The calibration of censored data is mainly visual. In this study, 60-months survival was used for the calibration of cox models, which corresponds to our median follow-up.

All analyses were performed in global population and after stratification by BC subtype. Qualitative variables were compared by Chi-square or Fisher exact tests and quantitative ones by Student *t*-tests. Survival probabilities were estimated by Kaplan–Meier method, and survival curves were compared with log-rank tests. Hazard ratios and their 95% confidence intervals were calculated with the Cox proportional hazards model. Significance threshold was of 5%. Analyses were performed with R software, version 3.3.^30^

## Results

### Patient characteristics

Our cohort was composed of 750 patients. Patient characteristics are detailed in Appendix Table A1. Mean age at diagnosis was of 48.3 years. Tumour distribution by BC subtype was as follows: luminal: *n* = 221 (29.5%); TNBC: *n* = 320 (42.7%); *HER2-*positive: *n* = 209 (27.9%). At NAC completion, 281 (37.5%) patients had reached pCR. After a median follow-up of 52.8 months (CI [50.2–56.3]), 146 events were observed.

The distribution of patients into the three risk categories established is detailed Table 1. The distribution of RCB and Neo-Bioscore in global population and by BC subtype is represented in Appendix Fig. A1.

**Table 1:** Distribution of RCB and Neo-Bioscore, in global population and by BC subtype.

Scores	Global population	Luminal	TNBC	*HER2*-positive	Risk category
	*n* = 750	*n* = 221	*n* = 320	*n* = 209	
RCB					
RCB-0	281 (37.5)	31 (14)	138 (43.1)	112 (53.6)	Low (44.5%)
RCB-I	53 (7.1)	14 (6.3)	19 (5.9)	20 (9.6)	
RCB-II	286 (38.1)	100 (45.2)	123 (38.4)	63 (30.1)	Intermediate (38.1%)
RCB-III	130 (17.3)	76 (34.4)	40 (12.5)	14 (6.7)	High (17.3%)
Neo-Bioscore					
0	5 (0.7)	0 (0)	0 (0)	5 (2.4)	Low
1	54 (7.2)	12 (5.4)	0 (0)	42 (20.1)	(58.8%)
2	147 (19.6)	54 (24.4)	12 (3.8)	81 (38.8)	
3	235 (31.3)	79 (35.7)	96 (30)	60 (28.7)	
4	227 (30.3)	73 (33)	133 (41.6)	21 (10)	Intermediate
5	76 (10.1)	3 (1.4)	73 (22.8)	0 (0)	(40.4%)
6	6 (0.8)	0 (0)	6 (1.9)	0 (0)	High
7	0 (0)	0 (0)	0 (0)	0 (0)	(0.8%)

Low-risk category = predicted 5-year DFS > 90%, Intermediate-risk category = predicted 5-year DFS comprised between 70 and 90%, High-risk categor = predicted 5-year DFS < 70%.

According to RCB, 44.5% of patients were classified in the low-risk category (37.5% for RCB-0 and 7.1% RCB-I, respectively), 38.1% were classified in the intermediate-risk category and 17.3% were classified in the high-risk category.

According to Neo-Bioscore, 58.8% of patients were classified in the low-risk category (Neo-Bioscore 0/1/2/3: 1%; 8%; 19% and 32.7%, respectively), 40.4% were classified in the intermediate-risk category, whereas only 0.8% were classified in the high-risk category.

### Association between Neo-Bioscore, RCB and DFS

**Fig. 1:**
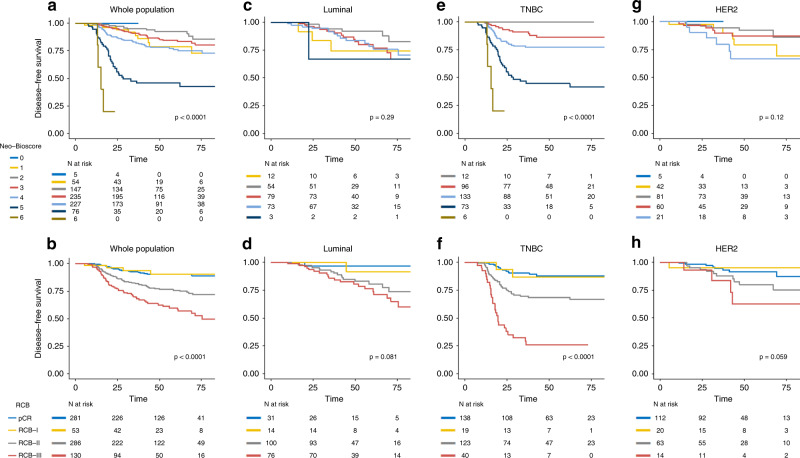
Association between RCB, Neo-Bioscore and disease free survival in the whole population and by pathological subtypes. **a** Neo-bioscore in the whole population; **b** RCB in the whole population; **c** Neo-bioscore in the Luminal subtype; **d** RCB in the Luminal subtype; **e** Neo-bioscore in the Triple negative subtype; **f** RCB in the Triple negative subtype; **g** Neo-bioscore in the *HER2*-positive subtype; **h** RCB in the *HER2*-positive subtype.

RCB and Neo-Bioscore were both significantly associated with DFS in global population (*p* < 0.0001) (Fig. 1a, b) and for TNBC patients (*p* < 0.0001) (Fig. 1e, f). A trend in favour of an association between RCB and DFS was observed for *HER2*-positive and luminal BC (*p* = 0.059 and *p* = 0.08, respectively) (Fig. 1c, d) (Fig. 1g, h).

RCB-0 and RCB-I curves were superimposed in all cases (in global population and in every BC subtype) and were both associated to a very good prognosis (5 y DFS rate = 90%, CI [86.5–94.4%] and 90% CI [81.7–100], respectively).

Similar results were obtained for OS (Appendix Fig. A2).

### Comparison of prognostic performance

#### Prognostic performance

**Fig. 2:**
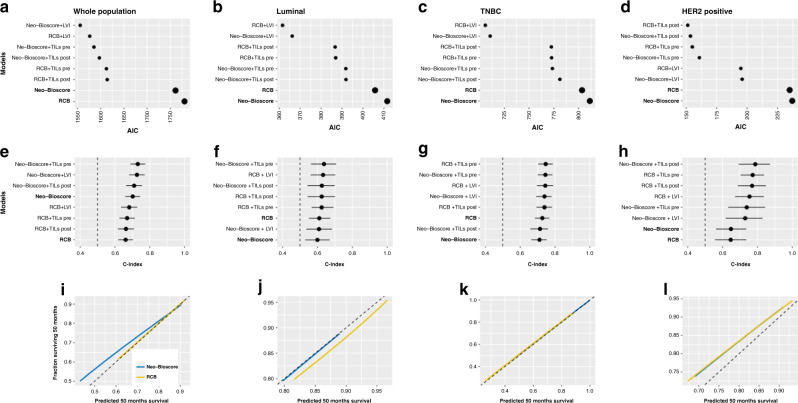
AIC, C-index and calibration curves for RCB and neo-bioscore, in whole population and by pathological substypes. **a** AIC in the whole population; **b** AIC in the luminal subtype; **c** AIC in the triple negative subtype; **d** AIC in the *HER2*-positive subtype; **e** C-index in the whole population; **f** C-index in the luminal subtype; **g** C-index in the triple negative subtype; **h** C-index in the *HER2*-positive subtype; **i** Calibration curves for the whole population; **j** Calibration curves for the luminal subtype; **k** Calibration curves for the triple negative subtype; **l** Calibration curves for the *HER2*-positive subtype.

We assessed the prognostic performance of Neo-Bioscore and RCB by calculating AIC (Fig. 2a–d). In global population, Neo-Bioscore was associated to a slightly lower AIC than RCB (AIC: 1738 vs. 1756, respectively), corresponding to a better prognostic performance.

However, RCB had the lowest AICs for every BC subtype (luminal: 403 vs. 408, respectively; TNBC: 800 vs. 808, respectively; *HER2*-positive BC: 232 vs. 234, respectively).

#### Discrimination

In global population, C-Index values were poor for RCB (0.66; CI [0.61–0.71]) and fair for Neo-Bioscore (0.70; CI [0.65–0.75]). C-Index values were higher in TNBC (RCB: 0.73, 9CI [0.68–0.77]; Neo-Bioscore: 0.71, CI [0.66–0.75]) and *HER2*-positive tumours (RCB: 0.64, CI [0.56–0.73]; Neo-Bioscore: 0.64, CI [0.56–0.73]) compared to luminal tumours (RCB: 0.61, CI [0.55–0.67], and Neo-Bioscore: 0.60, CI [0.53–0.67]) (Fig. 2e-h).

#### Calibration

Five-year calibration curves are represented Fig. 2i–l. Both Neo-Bioscore and RCB were well calibrated in global population and in every BC subtype. The best calibration was observed for TNBC patients. Altogether, both RCB and Neo-Bioscore were well calibrated among BC subtypes, and accurately discriminated the risk of patients. Neo-Bioscore had good performance in global population. RCB had slightly better prognostic performances when each BC subtype was evaluated separately.

### Concordance between Neo-Bioscore and RCB

**Fig. 3:**
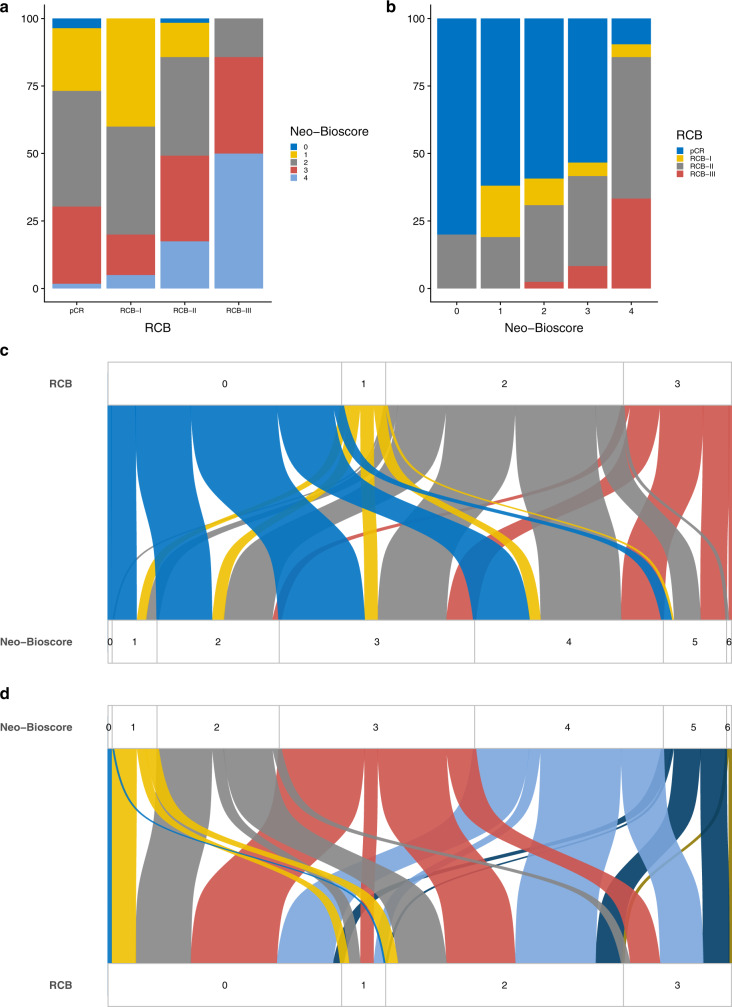
Concordance between RCB and neo-bioscore in the global population. **a** Neo-bioscore repartition according to RCB. **b** RCB repartition according to neo-bioscore. **c** Sankey plot of repartition of neo-bioscore according to RCB. **d** Sankey plot of repartition of RCB according to neo-bioscore.

Distributions of Neo-Bioscore according to RCB, and RCB according to Neo-Bioscore, respectively, are detailed Fig. 3. Concordance between the two scores was poor. 28% of patients classified RCB-pCR (i.e. low-risk category) corresponded to intermediate and high-risk categories according to Neo-Bioscore (Neo-Bioscore 4: 23.8%, and Neo-Bioscore 5: 4.2%, respectively) (Fig. 3a, c). Conversely, 34.6% patients classified RCB-III (i.e. high-risk category) corresponded to low-risk categories according to Neo-Bioscore (Neo-Bioscore 2–3: 7.7% and 26.9%, respectively).

Similarly, among the patients classified in the low-risk categories according to Neo-Bioscore (Neo-Bioscore 0–3), 49.8% corresponded to intermediate or high-risk categories according to RCB (RCB-II or III) (Fig. 3b, d).

Hence, on an individual scale, RCB and Neo-Bioscore displayed poor consistency (Fig. 3c). RCB and Neo-Bioscore were discrepant in 49.3% of cases when classifying patients into the three risk categories (low/intermediate/high). Despite good prognostic performances on a population scale, RCB and Neo-Bioscore were poorly concordant on an individual scale.

In BC subtypes, the highest concordance was observed for *HER2*-positive tumours (67% vs. only 30% of concordant predictions for luminal tumours) (Appendix Figs. A3, A4). 50.9% of predictions were concordant for TNBC tumours (Appendix Fig. A5). In the patient with a real event (recurrence, metastasis or death), concordance decreased to 35.5% in global population, 29.4% in *HER2*-positive tumours, and only 16.6% in the luminal tumours (vs. 48% in the TNBC population), underlying the difficulty of models to predict survival in high-risk groups.

### Addition of histological variables

Adding pathological variables to RCB and Neo-Bioscore slightly improved their prognostic performance.

In global population, AICs of models were systematically improved by adding LVI or pre-/post-NAC TILs level (Fig. 2). The best AIC was observed for Neo-Bioscore + LVI.

When analysing by BC subtype (Fig. 2), LVI also improved AICs of both RCB and Neo-Bioscore for TNBC and luminal tumours. For *HER2*-positive patients, pre- and post-NAC TILs levels improved models the most.

However, adding pathological variables did not improve C-Index, neither in global population, nor in BC subtypes.

## Discussion

Our study on a large cohort of BC patients treated with NAC showed that RCB and Neo-Bioscore were both significantly associated with prognosis in global population and in BC subtypes, especially for TNBC patients. Analyses were not relevant in the *HER2*-positive population due to the small number of events. Neo-Bioscore showed better performance in global population, whereas RCB offered better performance in pathological subtypes, notably for luminal and TNBC. In addition, our results suggest that these scores might be improved by the addition of pathological variables such as TILs or LVI. However, on an individual scale, RCB and Neo-Bioscore were highly discrepant in their predictions (~50% of consistency between risk categories).

Both scores had been individually validated in independent cohorts. An external validation of Neo-Bioscore was performed by Bergquist et al.^31^ on 43.320 patients from the National Cancer Database. Neo-Bioscore had a greater discriminative power compared to CPS + EG and AJCC clinical staging (5-year OS). The long-term prognostic relevance of RCB within each BC subtype was demonstrated by Symmans et al.^32^ in five BC cohorts (*n* = 1158). RCB has also been shown to be highly reproducible.^33^ Concerning direct comparison of the different scoring systems, Choi et al.^34^ evaluated seven pathologic classification systems, of which RCB and CPS + EG. RCB had the best performance compared to the other scoring systems, both for OS and distant disease-free survival (DDFS). However, Neo-Bioscore (an improvement of the CPS + EG score) was not evaluated in the latter study. Provenzano et al.^24^ had already suggested that RCB index was the preferred method for more detailed quantification of residual disease at NAC completion. Hence, our study adds strength to literature by being the first to our knowledge to compare the prognostic performance of RCB and Neo-Bioscore on a large real-life cohort of BC patients.

Identifying the best model to classify patients into prognostic categories after NAC is crucial, notably since scoring systems can help identify patients of poor prognosis that can be candidates to further second-line treatments or clinical trials. In addition, it appears important to determine the best prognostic score so that a unique score can be used in studies, to help improve their comparability. Indeed, different staging systems yield different estimates for future risks. Very different predictions can be obtained for the same patient. In our study, both scores showed high performance on a population scale, but were poorly concordant with one another on an individual scale. These limitations are common to numerous risk calculators and partially explains the lack of widespread use in routine practice.^35^ These differences could be explained by the fact that these models do not capture the same response patterns. Indeed, whereas RCB only considers tumour size variables (primary tumour dimensions, tumour bed cellularity and axillary nodal burden), Neo-Bioscore also comprises pathological characteristics such as oestrogen-receptor status, nuclear grade and *HER2* status. Altogether, since prognostic predictions in global population are currently outdated, pathological subtypes should be considered as distinct entities, and their prognosis should be evaluated independently. Another explanation for these discrepancies could come from the difference (and the very large number) of categories of both models. Hence, the worst concordance rates were found in the patient who expected an event (recurrence, death or metastasis), highlighting the difficulty of numerous models to identify the high-risk groups.

In an effort to improve prognostic performance, other studies suggested considering pathological variables as prognostic elements after NAC, alone or in combination with existing models.^13,20,23^Von Minckwitz et al.^36^ evaluated Ki67, a proliferation index, as a prognostic marker on 1151 patients after NAC. Patients with high Ki67 levels (>35.1) had significantly higher recurrence and death rates compared to patients with low or intermediate Ki67 levels. Adding Ki67 to the analysis of pCR was more contributive than pCR only for luminal BC. Sheri et al.^37^ also showed that the addition of post-treatment Ki67 to RCB improved the prediction of long-term outcome. However, Ki67 suffers from poor reproducibility and its assessment is difficult to standardise.^38^ Hence, its clinical utility remains limited in routine care. TILs on pretreatment biopsy have been associated with high pCR rates in the neoadjuvant setting and with better outcomes in the adjuvant setting.^14,20,21,23^ In addition, their assessment is rather standardised according to guidelines of the TILs working group.^27^ The added value of post-NAC TILs remains to be determined. Indeed, different prognostic values have been described among BC subtypes, with higher levels being associated with a good outcome in TNBC patients with residual disease after NAC,^39^ but with a poor outcome in *HER2*-positive patients.^23^ Asano et al.^40^ suggested that RCB combined with post-NAC TILs could be a more sensitive predictor for BC recurrence after NAC, with a 0.048 risk of recurrence (hazard ratio) in global population for RCB-TILs-positive patients (*p* < 0.001), 0.041 in TNBC patients (*p* = 0.018), 0.134 in *HER2*-positive patients (*p* = 0.036) and 0.081 for luminal tumours (*p* = 0.002). Future models should probably integrate this information.

In conclusion, our results suggest that although RCB and Neo-Bioscore offer similar prognostic performances to classify patients at NAC completion in global population, RCB showed better performance in pathological subtypes, especially in luminal and TNBC. RCB offers the advantage of generating fewer prognostic classes compared to Neo-Bioscore, which enables an easier use in everyday real-life practice. Further studies are warranted to confirm the present data and to evaluate the prognostic performance of other pathological variables like TILs or LVI that could be included in future scores to improve their prognosis performance.

## Supplementary information

Supplemental Material

## Data Availability

All supplementary data are available from authors on reasonable request.
