# Supplementary material for: Determination of breast cancer prognosis after neoadjuvant chemotherapy: comparison of Residual Cancer Burden (RCB) and Neo-Bioscore
Source: Br J Cancer. 2021 Feb 9;124(8):1421–7. doi: 10.1038/s41416-020-01251-3 (PMC8039034; doi:10.1038/s41416-020-01251-3)

**Supplemental: ER/PR/*HER2* positivity determination**

Histological grade was performed according to the Elston and Ellis (1991) modification of the Scarff–Bloom–Richardson grading system(1). Hormone receptors were analyzed by immunohistochemistry. ER and PR status were considered positive if 10% of carcinomatous cells stained positive, as recommended by French guidelines(2). *HER2* overexpression status was determined according to American Society of Clinical Oncology guidelines(3). Scores 3+ were reported as positive, score 1+/0 as negative. Tumors with scores 2+ were further tested by FISH. With regards to HER2 gene amplification, an average of 40 tumor cells per sample was evaluated and the mean HER2 signals per nuclei was calculated. A HER2/CEN17 ratio≥2 was considered positive and a ratio<2 negative.

1. Elston CW, Ellis IO. Pathological prognostic factors in breast cancer. I. The value of histological grade in breast cancer: experience from a large study with long-term follow-up. Histopathology. 1991 Nov;19(5):403–10.

2. Balaton AJ, Doussal VL, Arnould L, Barlier C, Bellocq JP, Ettore F, et al. Recommandations pour l’évaluation immunohistochimique des récepteurs hormonaux sur coupes en paraffine dans les carcinomes mammaires Mise à jour 1999. /data/revues/02426498/00190004/336/ [Internet]. 2008 Feb 20 [cited 2019 Jun 6]; Available from: https://www.em-consulte.com/en/article/88258

3. Wolff A. Recommendations for human epidermal growth factor receptor 2 testing in breast cancer: American Society of Clinical Oncology/College of American Pathologists clinical practice guideline update. Journal of Clinical Oncology. 2013 Nov 1;3997–4013.

**Supplemental Table 1: Patient characteristics, in global population and by pathological subtype**

| Variables | **Whole population**  n= 750 | **Luminal**  n= 221 | **TNBC**  n= 320 | ***HER2-*positive**  n= 209 | *p*-value |
| --- | --- | --- | --- | --- | --- |
|  |  |  |  |  |  |
| Age (years)  <45  45-55  >55 | 293 (39.1)  265 (35.3)  192 (25.6) | 90 (40.7)  81 (36.7)  50 (22.6) | 122 (38.1)  117 (36.6)  81 (25.3) | 81 (38.8)  67 (32.1)  61 (29.2) | 0.085 |
| Menopausal | 278 (37.4) | 74 (33.8) | 120 (38) | 84 (40.4) | 0.42 |
| BMI  20≤BMI≤30  BMI<20  BMI>30 | 559 (74.8)  86 (11.5)  102 (13.7) | 154 (70)  31 (14.1)  35 (15.9) | 245 (76.8)  32 (10)  42 (13.2) | 160 (76.9)  23 (11.1)  25 (12) | 0.61 |
| Tumor size  ≤20 mm  >20 mm | 66 (8.8)  683 (91.2) | 15 (6.8)  205 (93.2) | 29 (9.1)  291 (90.9) | 22 (10.5)  187 (89.5) | 0.018 |
| T status  T1  T2  T3-4 | 50 (6.7)  504 (67.2)  196 (26.1) | 9 (4.1)  161 (72.9)  51 (23.1) | 29 (9.1)  206 (64.4)  85 (26.6) | 12 (5.7)  137 (65.6)  60 (28.7) | 0.12 |
| N status  N0  N1  N2-3 | 300 (40)  415 (55.3)  35 (4.7) | 78 (35.3)  134 (60.6)  9 (4.1) | 143 (44.7)  161 (50.3)  16 (5) | 79 (37.8)  120 (57.4)  10 (4.8) | 0.38 |
| Grade  I  II  III | 15 (2)  204 (27.8)  514 (70.1) | 10 (4.7)  105 (49.1)  99 (46.3) | 5 (1.6)  32 (10.2)  277 (88.2) | 0 (0)  67 (32.7)  138 (67.3) | <0.0001 |
| Mitotic Index  0-10  11-21  >22 | 190 (26.8)  208 (29.4)  310 (43.8) | 85 (41.3)  62 (30.1)  59 (28.6) | 49 (16.2)  72 (23.8)  182 (60.1) | 56 (28.1)  74 (37.2)  69 (34.7) | <0.0001 |
| Histology  Ductal  Lobular  Other | 691 (92.6)  23 (3.1)  32 (4.3) | 198 (89.6)  17 (7.7)  6 (2.7) | 292 (92.1)  4 (1.3)  21 (6.6) | 201 (96.6)  2 (1)  5 (2.4) | <0.0001 |
| pCR | 281 (37.5) | 31 (14) | 138 (43.1) | 112 (53.6) | <0.001 |
| Breast-conserving surgery | 547 (72.9) | 146 (66.1) | 250 (78.1) | 151 (72.2) | <0.0001 |
| Radiotherapy | 750 (100) | 221 (100) | 320 (100) | 165 (100) | <0.0001 |
| Hormone therapy | 311 (41.4) | 212 (96.0) | 0 (0) | 98 (46.9) | <0.0001 |

**Supplemental Figure 1: Disease-free survival for HER2/ER+ and HER2/ER- patients**


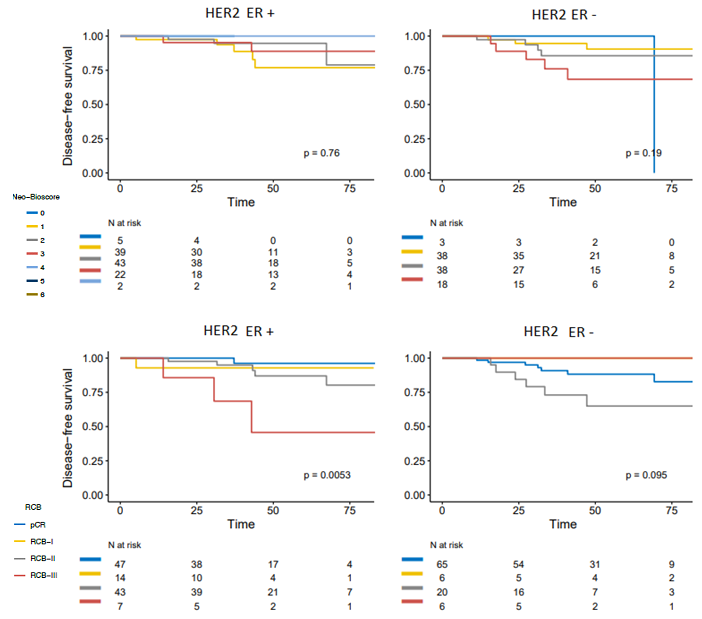


**Supplemental Figure 2: Association between RCB, Neo-Bioscore and overall survival**


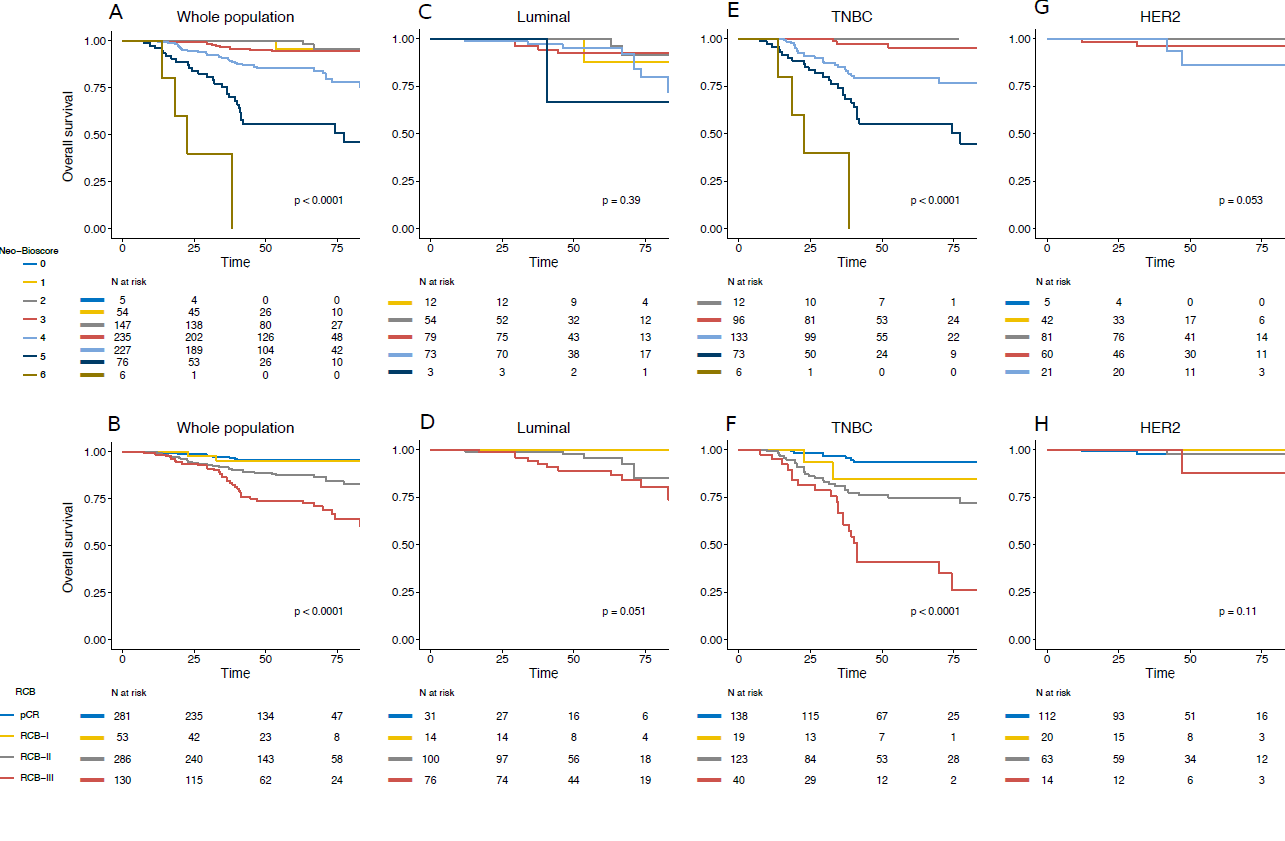


**Supplemental Figure 3: Overall survival for HER2/ER+ and HER/ER- patients**


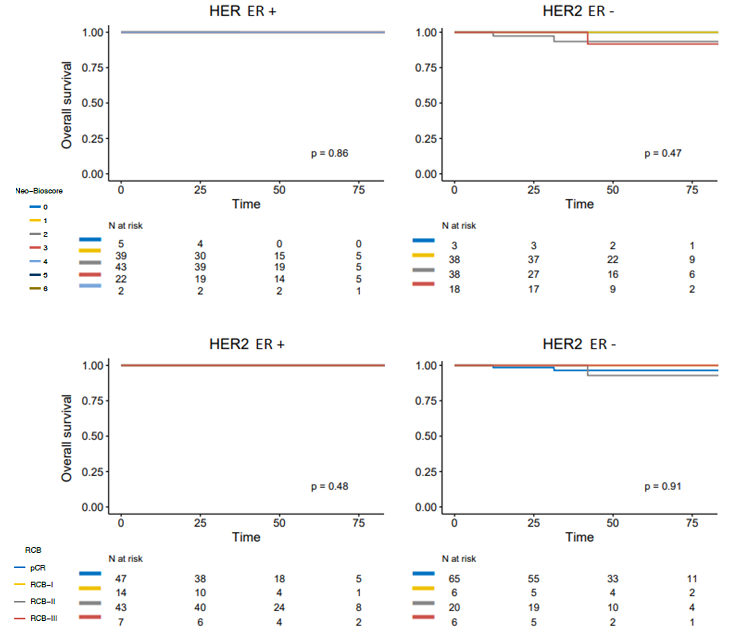


**Supplemental Figure 4: AIC, C-Index and calibration curves for RCB and Neo-Bioscore, for HER2/ER+ and HER/ER- patients**


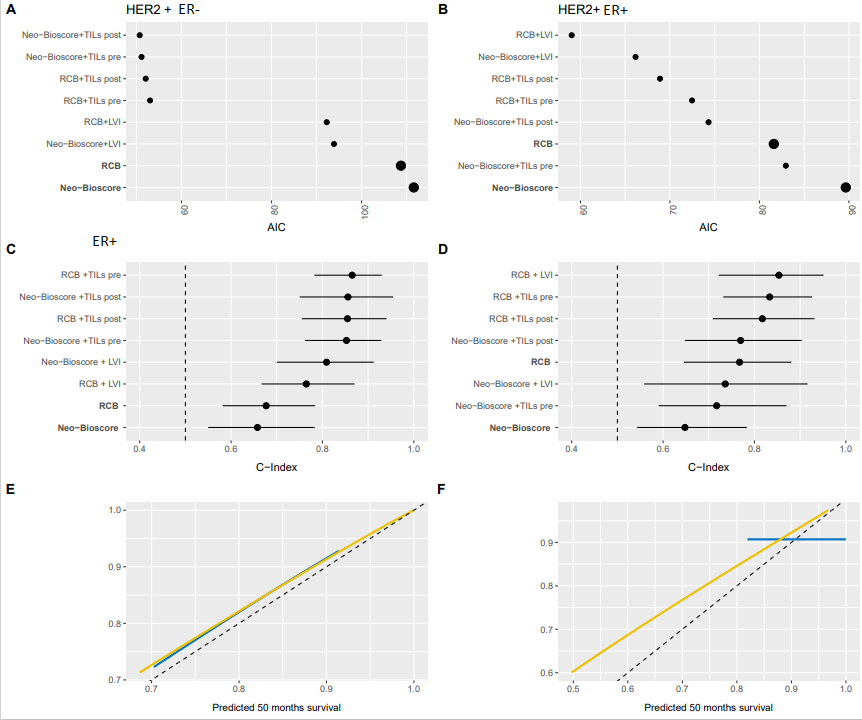


**Supplemental Figure 5: Concordance between RCB and Neo-Bioscore in HER2-positive tumors**


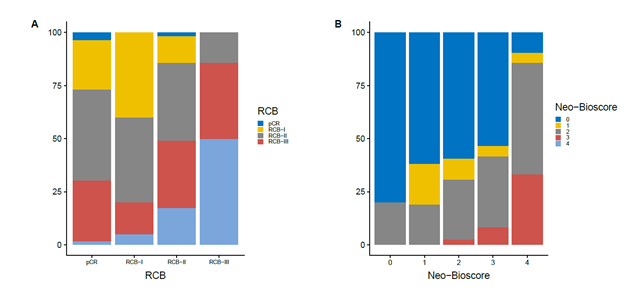


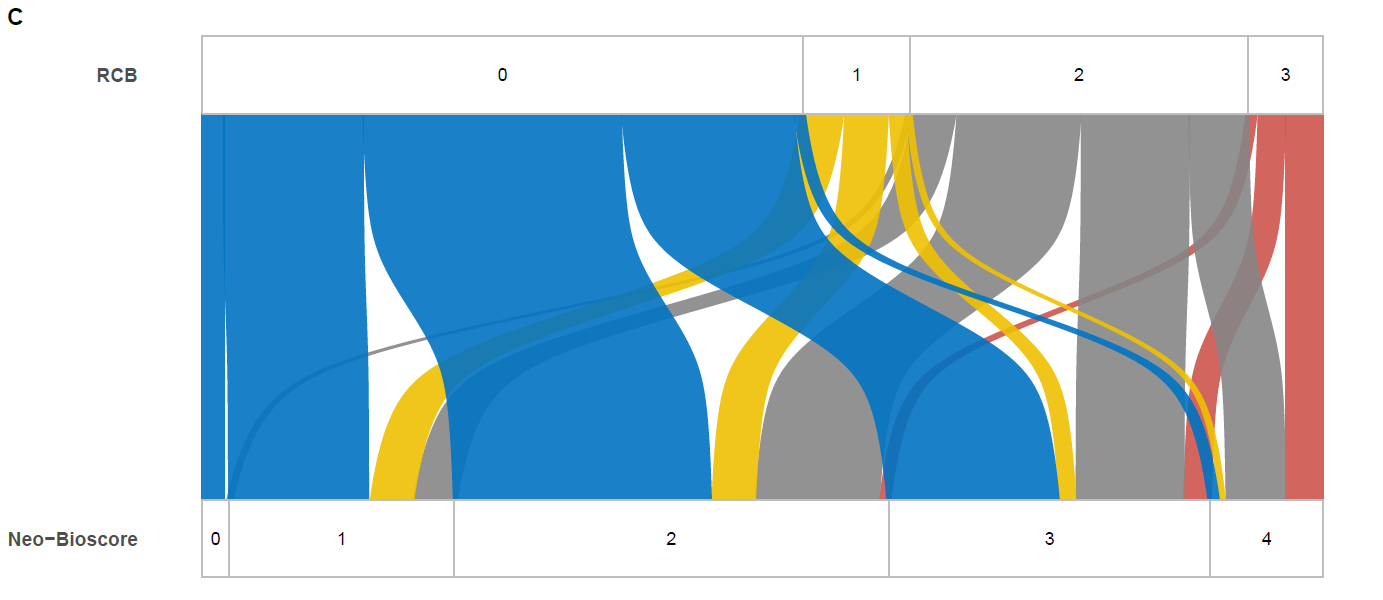


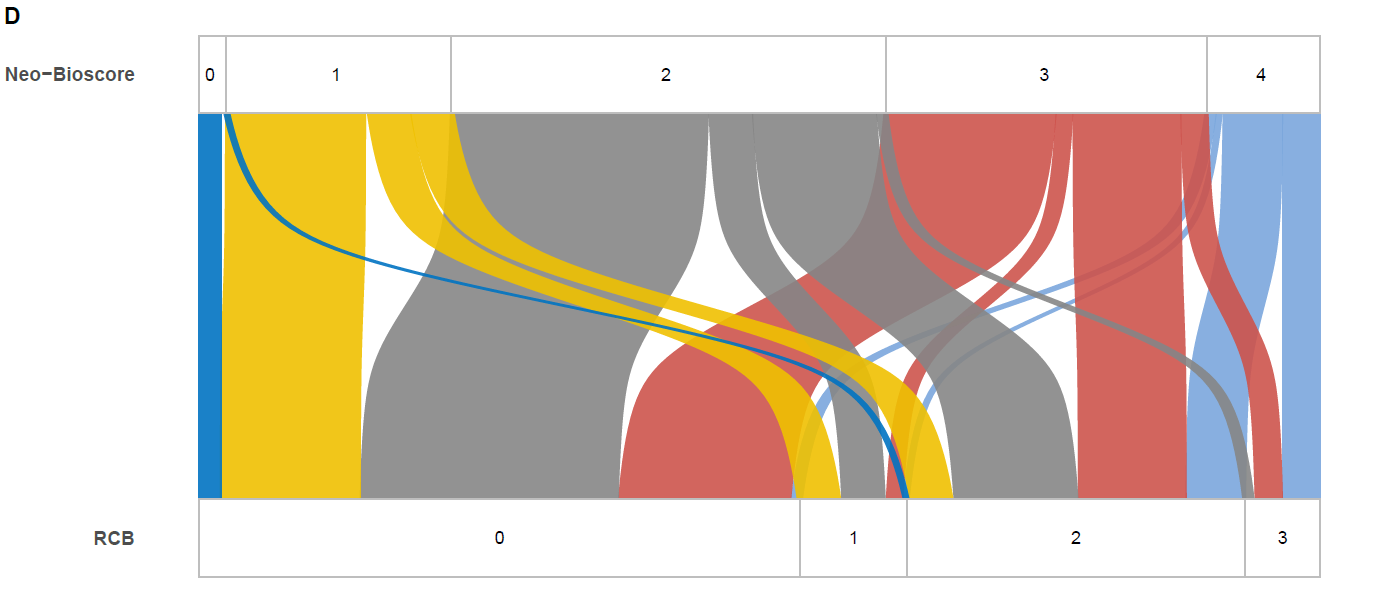


**Supplemental Figure 6: Concordance between RCB and Neo-Bioscore in luminal tumors**


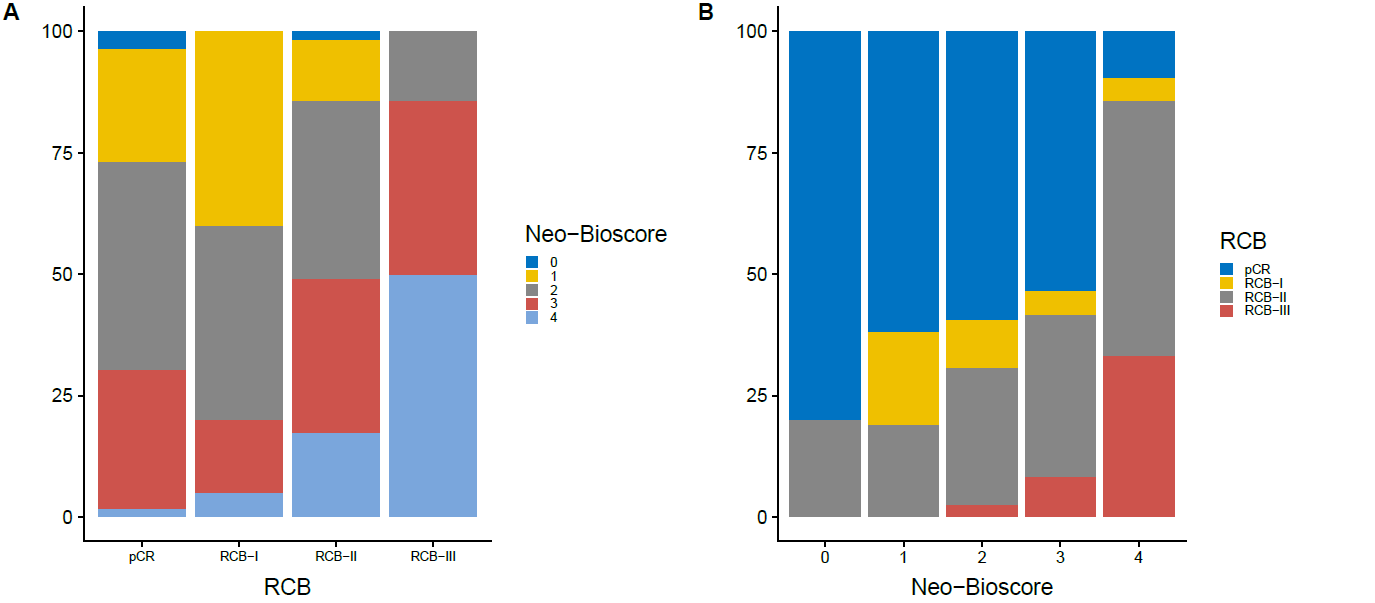


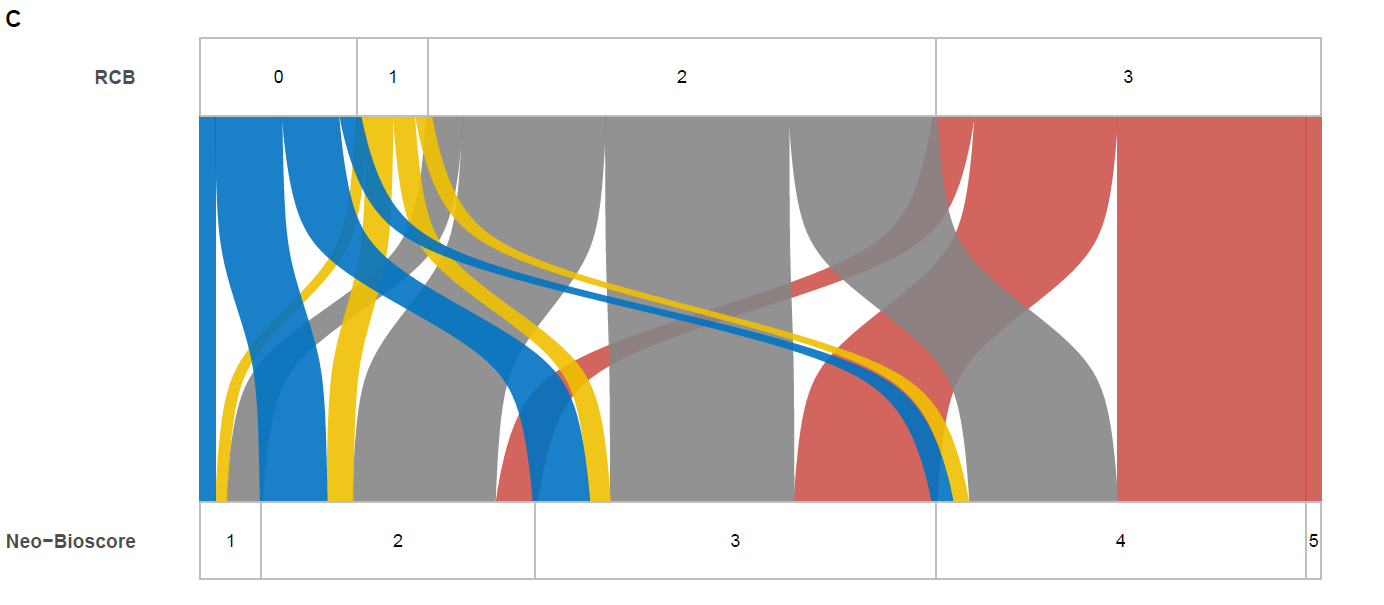


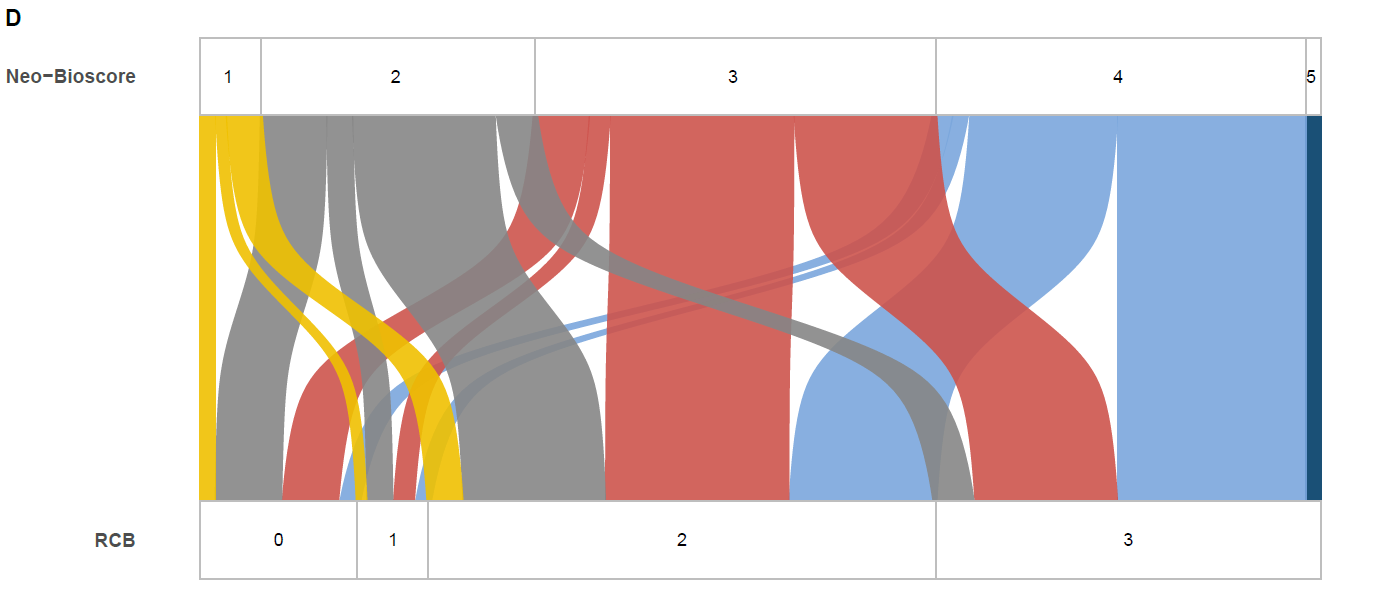


**Supplemental Figure 7: Concordance between RCB and Neo-Bioscore in TNBC**


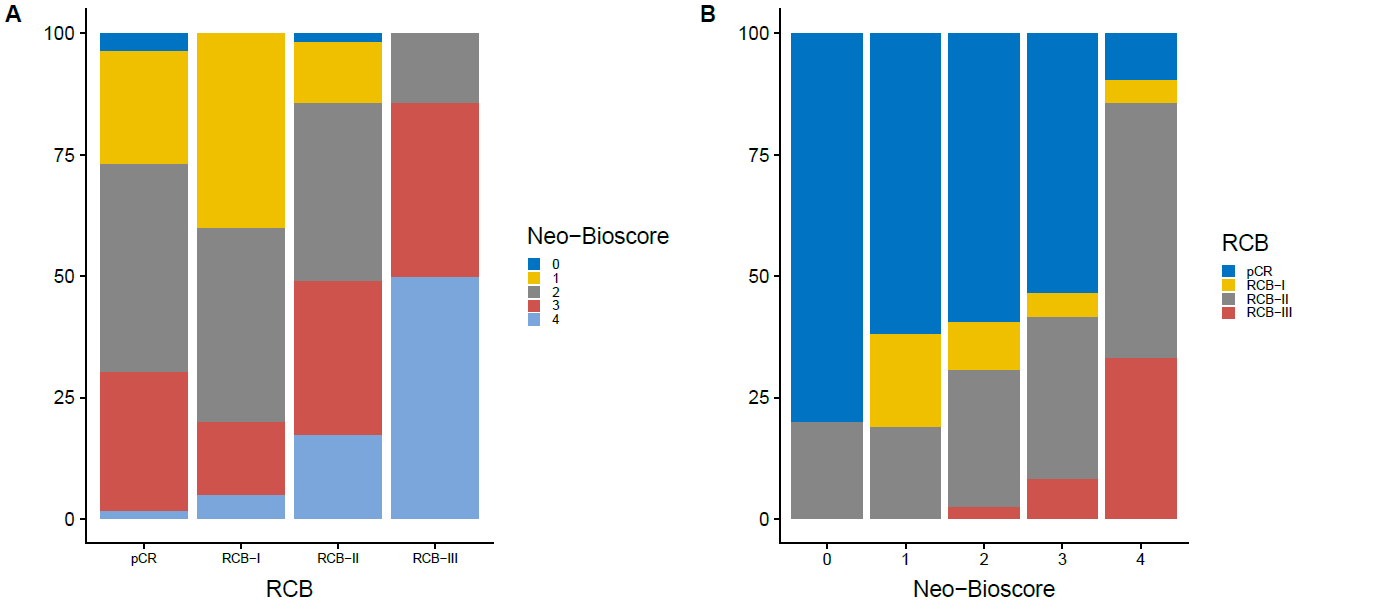


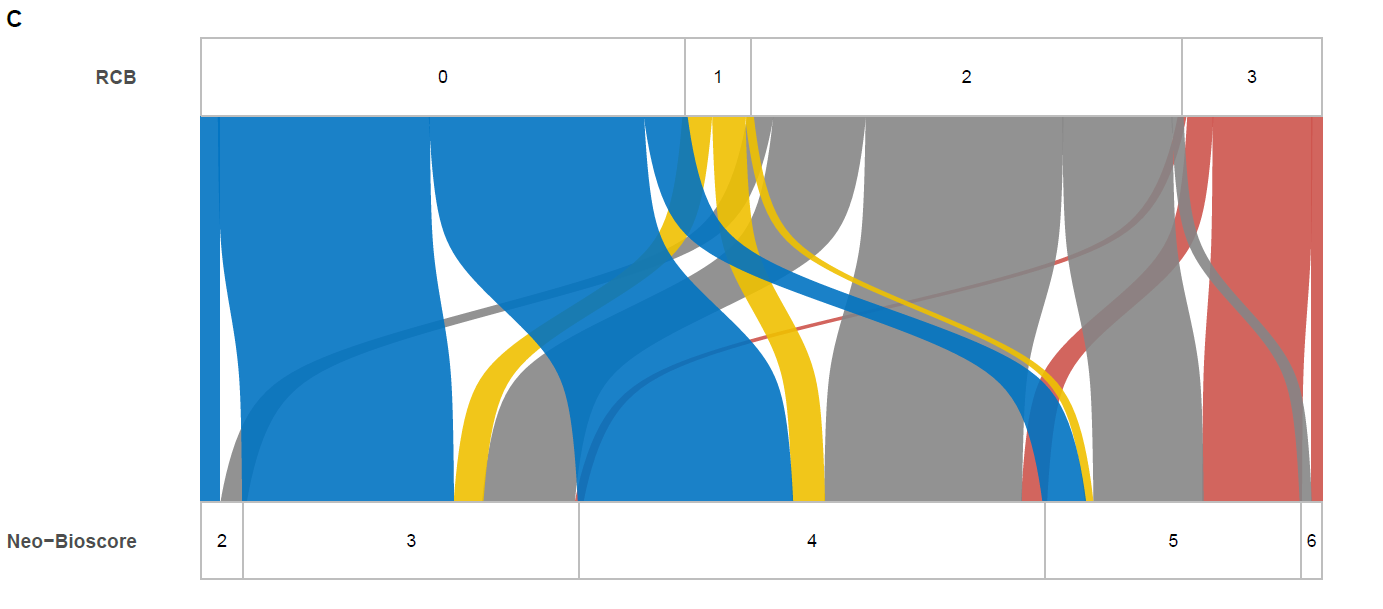


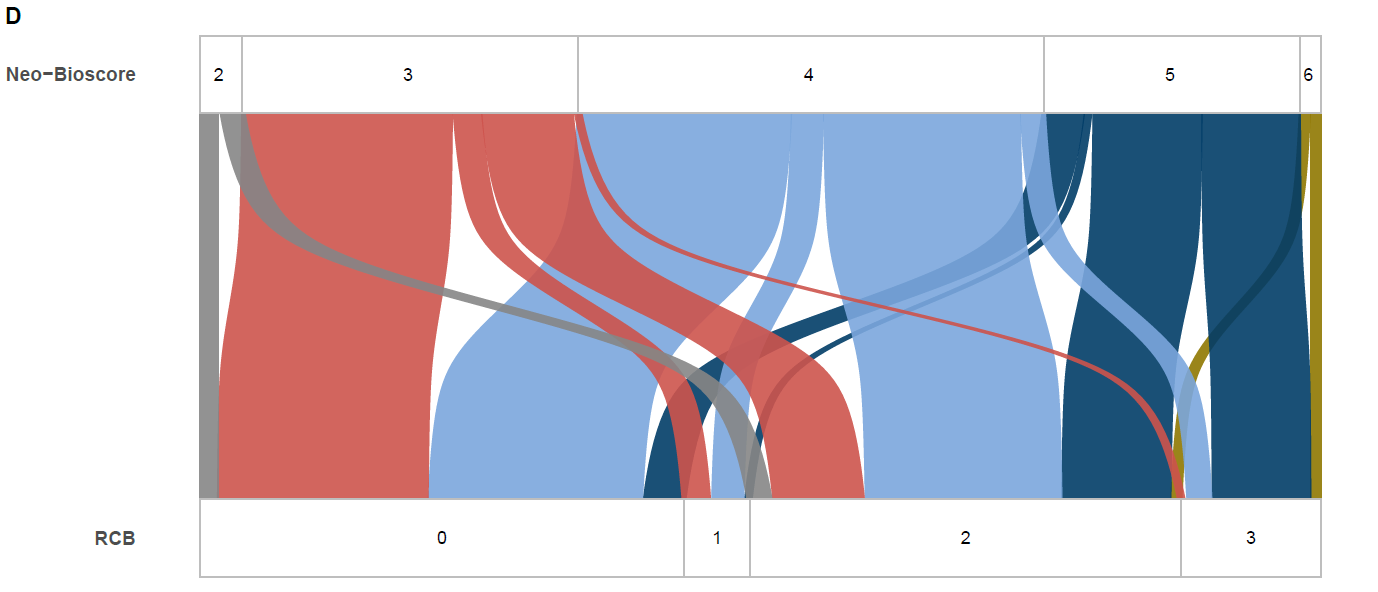


**Supplemental Figure 8: Distribution of stromal TILs and LVI according to BC subtype**


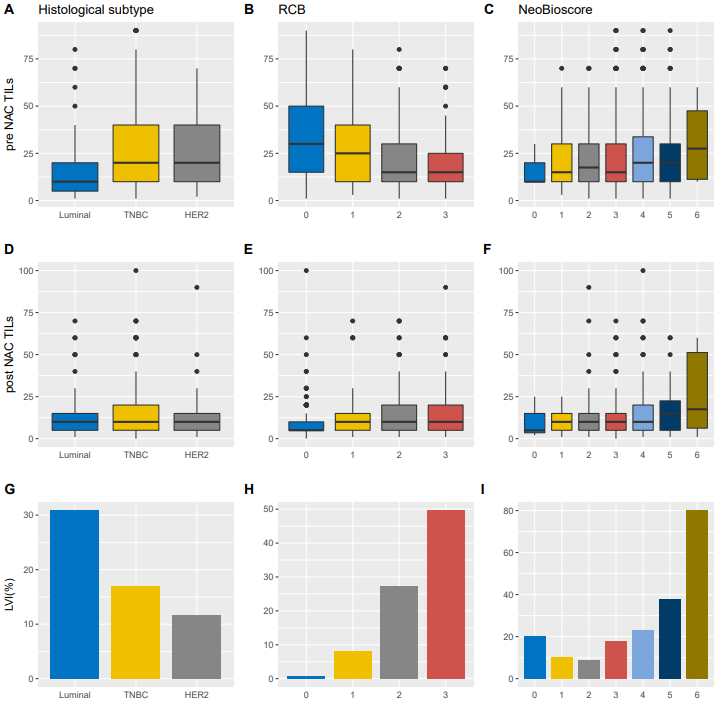

Supplement: Supplementary file 1 — Supplemental Material [file 41416_2020_1251_MOESM1_ESM.docx]
